# A comprehensive review of the family of very-long-chain fatty acid elongases: structure, function, and implications in physiology and pathology

**DOI:** 10.1186/s40001-023-01523-7

**Published:** 2023-11-20

**Authors:** Xiangyu Wang, Hao Yu, Rong Gao, Ming Liu, Wenli Xie

**Affiliations:** 1 Department of Gynecological Oncology, Shandong Cancer Hospital and Institute, Shandong First Medical University and Shandong Academy of Medical Sciences, Jinan, Shandong 250117 People’s Republic of China; 2 Department of Gynecology, The Second Hospital of Shandong University, Jinan, Shandong 250033 People’s Republic of China

**Keywords:** Very-long-chain fatty acid elongases, Cellular physiology, Metabolic disorders, Cancer, Personalized medicine

## Abstract

**Background:**

The very-long-chain fatty acid elongase (ELOVL) family plays essential roles in lipid metabolism and cellular functions. This comprehensive review explores the structural characteristics, functional properties, and physiological significance of individual ELOVL isoforms, providing insights into lipid biosynthesis, cell membrane dynamics, and signaling pathways.

**Aim of review:**

This review aims to highlight the significance of the ELOVL family in normal physiology and disease development. By synthesizing current knowledge, we underscore the relevance of ELOVLs as potential therapeutic targets.

**Key scientific concepts of review:**

We emphasize the association between dysregulated ELOVL expression and diseases, including metabolic disorders, skin diseases, neurodegenerative conditions, and cancer. The intricate involvement of ELOVLs in cancer biology, from tumor initiation to metastasis, highlights their potential as targets for anticancer therapies. Additionally, we discuss the prospects of using isoform-specific inhibitors and activators for metabolic disorders and cancer treatment. The identification of ELOVL-based biomarkers may advance diagnostics and personalized medicine.

**Conclusion:**

The ELOVL family's multifaceted roles in lipid metabolism and cellular physiology underscore its importance in health and disease. Understanding their functions offers potential therapeutic avenues and personalized treatments.

## Introduction

The family of very-long-chain fatty acid elongases (ELOVL1-ELOVL7) plays a crucial role in cellular metabolism by facilitating the elongation of fatty acids beyond the typical chain length. These elongases are responsible for synthesizing very-long-chain fatty acids (VLCFAs), which are fatty acids with carbon chain lengths of 20 carbons or more [22]. VLCFAs are essential components of various cellular processes, including lipid metabolism, membrane structure, and cell signaling [26, 27].

The significance of the very-long-chain fatty acid elongase (ELOVL) family is evident in its involvement in diverse physiological functions. These elongases are responsible for the synthesis of VLCFAs, which serve as precursors for various lipid species, such as ceramides, sphingolipids, and cholesterol esters [52]. Through their involvement in lipid synthesis and modification, the elongases influence vital cellular processes, including membrane composition and fluidity, lipid droplet formation, and lipid signaling pathways.

Moreover, emerging research has highlighted the association between alterations in the very-long-chain fatty acid elongase family and several diseases. Genetic mutations or dysregulation in the expression and activity of elongases have been implicated in various metabolic disorders, such as X-linked adrenoleukodystrophy (X-ALD), obesity, type 2 diabetes, and cardiovascular diseases [10, 32, 34]. Additionally, aberrant elongase function has been linked to skin disorders and cancer [15, 33].

The purpose of this review is to provide a comprehensive overview of the family of very-long-chain fatty acid elongases, their structure, function, and their roles in physiology and pathology. By synthesizing and analyzing the existing literature, this review aims to enhance our understanding of the significance and mechanisms of very-long-chain fatty acid elongases in cellular processes, as well as their implications in various diseases.

## Overview of the superfamily of very-long-chain fatty acid elongases

The family of very-long-chain fatty acid elongases consists of several members, each with distinct functions and characteristics. These family members are named and classified based on their specific roles in fatty acid elongation. The naming convention typically includes the designation "ELOVL" (Elongation of Very Long-chain fatty acids) followed by a number that denotes the specific elongase isoform.

For example, ELOVL1, ELOVL2, ELOVL3, ELOVL4, and ELOVL5 are among the well-studied members of the family. The classification of these elongases is based on their specific enzymatic activities and substrate preferences [24]. Some elongases are involved in the elongation of a wide range of fatty acids, while others exhibit substrate specificity for certain fatty acid chain lengths or types.

**Fig. 1:**
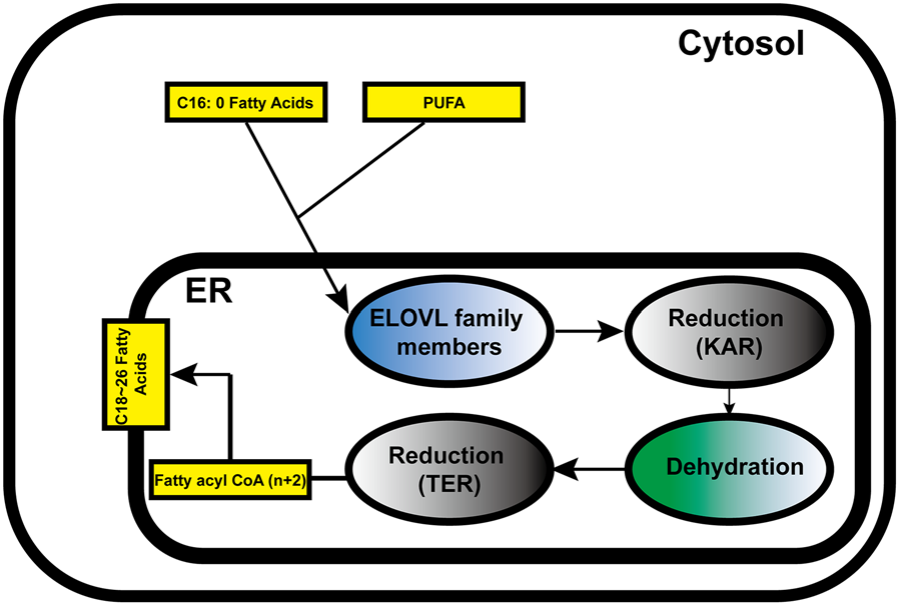
ELOVL Family Members in Cells. The ELOVL enzymes, short for "Elongation of Very Long Chain Fatty Acids," are the condensing enzymes that are proposed to govern substrate specificity and act as the bottleneck in the elongation process. KAR, 3-ketoacyl-CoA reductase; TER, trans-2,3-enoyl-CoA reductase; ER, endoplasmic reticulum

The members of the family of very-long-chain fatty acid elongases share common structural features and functional characteristics. These enzymes are integral membrane proteins that reside in the endoplasmic reticulum (ER) and function as catalysts in the fatty acid elongation process. Structurally, very-long-chain fatty acid elongases consist of transmembrane domains that anchor them to the ER membrane, as well as catalytic domains that facilitate the enzymatic reactions. These catalytic domains typically contain conserved motifs and residues essential for substrate binding and enzymatic activity [42]. Functionally, very-long-chain fatty acid elongases play a key role in the elongation of fatty acids by adding two carbon units to the acyl chain. They utilize specific enzymes and cofactors to catalyze the reactions, such as fatty acyl-CoA substrates and NADPH as a reducing agent [22]. These elongases are involved in the stepwise addition of carbon atoms to the growing fatty acid chain, ultimately producing very-long-chain fatty acids. Furthermore, each elongase isoform exhibits distinct substrate specificity and preference for fatty acid chain lengths (Fig. 1). For instance, ELOVL1 is known to preferentially elongate C12-C16 fatty acids, while ELOVL2 is involved in the elongation of polyunsaturated fatty acids, such as docosahexaenoic acid (DHA) [8, 45]. Understanding the structural and functional characteristics of these elongases provides insights into their roles in lipid metabolism and cellular processes.

ELOVL1 and ELOVL6 expression is primarily regulated through lipogenic mechanisms akin to fatty acid synthase (FAS), under the influence of factors such as liver X receptor (LXR) and sterol regulatory element-binding protein 1 (SREBP-1). Conversely, ELOVL3 expression is triggered by factors that promote fatty acid oxidation, such as norepinephrine (NE), while it is repressed by LXR. ELOVL2 and ELOVL5 both play a pivotal role in elongating polyunsaturated fatty acids (PUFAs), which, in turn, modulate the activity of peroxisome proliferator-activated receptor α (PPARα). Under specific conditions, like during periods of starvation, certain PUFAs, via PPARα, facilitate the synthesis of enzymes involved in fatty acid oxidation while suppressing lipogenesis through the active nuclear form of SREBP-1. Activation of PPARα has also been observed to induce the expression of ELOVL1, ELOVL3, ELOVL5, and ELOVL6, although not ELOVL2 and ELOVL4, which are regulated by peroxisome proliferator-activated receptor γ (PPARγ) on a longer-term basis. Additionally, Glucocorticoids (GC) are solely required for induced ELOVL3 expression. It's important to note that while ELOVL7 itself doesn't require NADPH for its activity, other components of the very-long-chain fatty acid (VLCFA) elongation machinery, 3-ketoacyl-CoA reductase (KAR) and trans-2,3-enoyl-CoA reductase (TER), which catalyze the second and fourth steps, respectively, utilize NADPH as a cofactor. Consequently, the progression of the entire VLCFA elongation cycle appears to enhance ELOVL7 activity [22](Table 1).

**Table 1:** Regulation of ELOVL family members in fatty acid metabolism

ELOVL family member	Regulation mode	Regulating factors
ELOVL1, ELOVL6	Lipogenic fashion similar to FAS	LXR, SREBP-1
ELOVL1, ELOVL3, ELOVL5, ELOVL6	Stimulated by PPARα	PPARα
ELOVL2	Controls elongation of PUFAs	Regulates the activity of PPARα
ELOVL3	Requires GC for induction	GC
ELOVL4	Regulated by PPARγ	PPARγ
ELOVL5	Controls elongation of PUFAs	Regulates the activity of PPARα
ELOVL7	Enhanced by progression of the entire VLCFA elongation cycle	Elongase complex built by the components of the VLCFA elongation machinery

FAS, fatty acid synthase; LXR, liver X receptor; SREBP-1, sterol regulatory element-binding protein 1; PPARα, peroxisome proliferator-activated receptor α; GC, glucocorticoids; PUFAs, polyunsaturated fatty acids; VLCFA, very-long-chain fatty acid

The expression of very-long-chain fatty acid elongase family members is also regulated in a tissue-specific manner. Different tissues and cell types exhibit varying expression patterns of these elongases, suggesting their involvement in specific physiological processes and functions. For example, ELOVL1 is highly expressed in the skin, where it plays a crucial role in maintaining the skin barrier function and sphingolipid levels [20]. ELOVL2 shows widespread expression in various tissues, including the brain, liver, and adipose tissue, indicating its involvement in multiple metabolic pathways happening in distinct tissues [57]. ELOVL3 was initially identified as an mRNA species that exhibited significant elevation in brown adipose tissue (BAT) from mice exposed to cold temperatures, a key process during BAT recruitment. Subsequent prolonged exposure to cold conditions over one month led to a gradual decrease in the heightened expression of ELOVL3, although it still remained significantly elevated compared to control levels [21]. ELOVL4 is predominantly expressed in the retina and is essential for the synthesis of very-long-chain polyunsaturated fatty acids required for proper vision [2]. ELOVL5 expression is detectable across various human tissues, with the highest mRNA levels observed in the testis and epididymis, in line with the presence of elevated docosapentanoic acid (22:5n–6), a polyunsaturated fatty acid (PUFA) metabolite, in these two tissues [6]. ELOVL6 exhibits widespread expression, particularly in tissues rich in lipids, such as BAT, white adipose tissue (WAT), and liver [18]. ELOVL7 mRNA is found in most tested tissues, except for the heart and skeletal muscle. Notably, ELOVL7 demonstrates high expression in the pancreas, kidney, prostate, and colon, while its expression is comparatively low in the lung, ovary, spleen, and thymus [45] (Table 2). The tissue-specific expression of very-long-chain fatty acid elongases is regulated by various factors, including transcriptional regulation, hormonal signaling, and developmental cues [5, 39, 64]. Elucidating the tissue-specific expression patterns and regulatory mechanisms provides valuable insights into the physiological roles and significance of these elongases in different tissues and cell types.

**Table 2:** Tissue-specific expression patterns of ELOVL family members and key roles in lipid metabolism

ELOVL family member	Tissue expression	Notable tissues & functions	References
ELOVL1	Highly expressed in the skin	Maintains skin barrier function and sphingolipid levels	Isokawa et al. [20]
ELOVL2	Widespread expression in various tissues	Brain, liver, adipose tissue; involvement in multiple metabolic pathways	Slieker et al. [57]
ELOVL3	Highly elevated in BAT	Involved in BAT recruitment, prolonged cold exposure maintains elevated expression	Jörgensen et al. [21]
ELOVL4	Predominantly expressed in the retina	Essential for the synthesis of very-long-chain polyunsaturated fatty acids for proper vision	Barabas et al. [2]
ELOVL5	Detected in several human tissues	Highest levels in testis and testis and epididymis due to high docosapentanoic acid (22:5n–6)	Castellini et al. [6]
ELOVL6	Ubiquitously expressed, especially in lipid-rich tissues	Found in BAT, WAT, and liver	Iizuka et al. [18]
ELOVL7	Expressed in most tissues except heart and skeletal muscle	High in pancreas, kidney, prostate, colon; low in lung, ovary, spleen, thymus	Ohno et al. [45]

BAT, brown adipose tissue; WAT, white adipose tissue

By understanding the naming and classification, as well as the structural, functional, and tissue expression characteristics of the superfamily of very-long-chain fatty acid elongases, we can gain a comprehensive overview of these enzymes and their contributions to cellular metabolism and lipid homeostasis.

## The role of the ELOVL family in physiological processes

### Role in lipid synthesis and modification

The ELOVL family plays a crucial role in lipid synthesis and modification. By catalyzing the elongation of very-long-chain fatty acids, these enzymes contribute to the production of a diverse array of lipids with specific functions in cellular processes.

#### Fatty acid synthesis

Fatty acids play a fundamental role in cellular metabolism, serving as key components in the synthesis of diverse lipid species that are vital for numerous cellular processes [9]. The ELOVL family is intricately involved in the synthesis of both long-chain and very-long-chain fatty acids, which are crucial building blocks for various lipids with essential functions in cells.

ELOVL enzymes are responsible for extending the carbon chain of fatty acids, thereby elongating them to form long-chain and very-long-chain fatty acids. These elongated fatty acids are crucial for the production of various lipid molecules, including triglycerides and phospholipids [4]. Triglycerides serve as a major energy reservoir in cells, facilitating the storage and release of energy as needed. During periods of energy excess, fatty acids are esterified to glycerol, forming triglycerides that are stored in specialized cellular compartments known as lipid droplets [55]. When energy demands increase, triglycerides are hydrolyzed back into fatty acids, which can be utilized as an energy source. Phospholipids, another important class of lipids, are integral components of cellular membranes. The fluidity and integrity of cellular membranes are essential for cell function and viability, and these properties are influenced by the composition of phospholipids [47]. ELOVLs contribute to the synthesis of very-long-chain fatty acids that are incorporated into phospholipids, influencing membrane fluidity and stability.

Moreover, certain fatty acids produced by ELOVL enzymes also serve as precursors for the synthesis of bioactive lipid signaling molecules. These signaling lipids, including various eicosanoids and docosanoids, play critical roles in cell signaling pathways, inflammation, and immune responses [48]. For instance, some fatty acids produced by ELOVLs can be converted into prostaglandins and leukotrienes, which are potent lipid mediators involved in various physiological and pathological processes, such as inflammation and pain perception [12].

In conclusion, ELOVL enzymes play a central role in fatty acid synthesis, contributing to the generation of long-chain and very-long-chain fatty acids that are essential for the production of various lipids with diverse functions in cells. The balanced and regulated activity of ELOVLs is crucial for maintaining cellular lipid homeostasis and normal physiological processes. Understanding the precise roles of ELOVLs in lipid metabolism and their implications in disease pathogenesis may pave the way for potential therapeutic interventions targeting these enzymes in various disorders.

#### Esterification

In addition to elongating fatty acids to form long-chain and very-long-chain fatty acids, the enzymatic activity of ELOVLs also plays an indirect role in the esterification of fatty acids with other molecules, leading to the generation of a wide range of lipid species with unique properties and functions.

One of the essential esterification reactions involving ELOVLs is the esterification of fatty acids with coenzyme A (CoA) to form fatty acyl-CoA molecules. Fatty acyl-CoAs are crucial intermediates in various metabolic pathways, including fatty acid oxidation and synthesis of complex lipids. For instance, fatty acyl-CoAs serve as substrates for the synthesis of triglycerides, phospholipids, and other complex lipids, which are important for energy storage, membrane structure, and cell signaling [60].

Furthermore, ELOVLs are involved in the esterification of fatty acids with glycerol to generate triglycerides, which represent a major form of energy storage in cells. The synthesis of triglycerides is vital for energy balance and involves the esterification of three fatty acyl groups to a glycerol backbone, forming a neutral lipid that can be stored in lipid droplets for later utilization during times of energy demand [63].

Moreover, ELOVL enzymes also participate in the esterification of fatty acids with cholesterol to produce cholesterol esters [62]. Cholesterol esters are critical for cellular cholesterol homeostasis, as they facilitate the storage and transport of cholesterol within cells and tissues. The regulation of cholesterol levels is crucial for maintaining membrane integrity, modulating cellular signaling pathways, and serving as a precursor for the synthesis of steroid hormones and bile acids [30].

Overall, the esterification and lipid modification activities of ELOVLs contribute to the production of a wide variety of lipid species with essential functions in cellular metabolism and physiology. The balanced regulation of these enzymatic activities is critical for maintaining lipid homeostasis and cellular functions. Dysregulation of ELOVL-mediated lipid modifications has been linked to various diseases, further highlighting the importance of understanding the precise roles of these enzymes in lipid metabolism and cellular processes. Targeting ELOVLs and their associated lipid pathways may hold promise for the development of novel therapeutic strategies for lipid-related disorders and beyond.

### Contribution to cellular membrane structure and function

The family of very-long-chain fatty acid elongases significantly impacts cellular membrane structure and function. By incorporating VLCFA into membrane lipids, these enzymes influence membrane properties and contribute to cellular homeostasis.

#### Membrane stability and fluidity

VLCFAs are known to increase membrane rigidity and stability. The elongases contribute to the incorporation of these fatty acids into membrane phospholipids, thereby influencing the fluidity and permeability of cellular membranes [25, 37].

#### Lipid rafts and microdomain formation

VLCFAs are crucial components of lipid rafts, specialized microdomains within cellular membranes. Lipid rafts play a role in organizing membrane proteins and facilitating various signaling processes [14]. The elongases contribute to the generation and maintenance of lipid rafts through the incorporation of VLCFAs.

### Regulatory role in cell signaling pathways

The ELOVL family also participates in the regulation of cell signaling pathways. Through the production of specific lipid species, these enzymes influence cellular signaling events and modulate various physiological processes.

#### Cell proliferation and survival

VLCFAs and their derivatives have been implicated in the regulation of cell proliferation and cell survival pathways. These lipids may modulate key regulatory factors involved in cell cycle control, thereby influencing cell proliferation and differentiation [50].

#### Signal transduction pathways

Very-long-chain fatty acid-derived lipids, such as sphingolipids and ceramides, participate in various signal transduction pathways. These lipids can act as signaling molecules or modulators of protein function, thereby regulating cellular responses to extracellular stimuli [19]. Bioinformatic analyses suggest that ELOVL1 may play a role in pathways related to the immune system. In the context of hepatocellular carcinoma (HCC), ELOVL1 appears to influence immune cell infiltration and the expression of immune checkpoint markers, such as programmed cell death-1 (PD-1) and cytotoxic T lymphocyte-associated protein-4 (CTLA-4) [69]. Impaired function of ELOVL2 disrupts lipid synthesis, leading to increased endoplasmic reticulum stress and mitochondrial dysfunction. This, in turn, activates key pathways associated with aging. Interestingly, overexpression of ELOVL2 can reverse these effects [28]. ELOVL3 is generally involved downstream in the MAPK signaling pathways, mTOR signaling pathways, and Wnt signaling pathways [65]. ELOVL4 regulates the synthesis of very long-chain (≥ C28) polyunsaturated fatty acids, including n-3 VLC-PUFAs, and has been implicated in neuroprotective signaling for maintaining photoreceptor cell integrity [68]. The use of CRISPR/Cas9-mediated knockout to target ELOVL5 inhibits AKT Ser473 phosphorylation and suppresses renal cancer cell invasion by downregulating chemokine (C–C motif) ligand-2 through the AKT-mTOR-STAT3 signaling pathway [44]. Studies have shown alterations in the expression and activity of ELOVLs in myelin-induced foam cells, with a particular focus on ELOVL6. ELOVL6, responsible for converting saturated and monounsaturated C16 fatty acids into C18 species, is upregulated in myelin phagocytosing phagocytes in vitro and in multiple sclerosis (MS) lesions. Depletion of ELOVL6 induces a repair-promoting phagocyte phenotype through the activation of the S1P/PPARγ pathway (Garcia Corrales, Verberk, & Haidar, 2023). Furthermore, research indicates differential expression of ELOVL7 in C2C12 cells under heat or cold stress. This differential expression is associated with enriched KEGG pathways, including the PI3K-Akt signaling pathway, lysosome, HIF-signaling pathway, Wnt signaling pathway, and AMPK signaling pathway [49] (Table 3).

**Table 3:** Functional roles of ELOVL family members in cellular pathways and diseases

ELOVL family member	Associated pathways and functions	References
ELOVL1	Affects immune cell infiltration, immune checkpoint markers such as PD-1 and CTLA-4 in HCC	Zhang et al. [69]
ELOVL2	Associated with key aging-associated pathways	Li et al. [28]
ELOVL3	Plays roles downstream of MAPK, mTOR, and Wnt signaling pathways	Wei et al. [65]
ELOVL4	Involved in neuroprotective signaling for photoreceptor cell integrity	Yeboah et al. [68]
ELOVL5	Knockout inhibits AKT Ser473 phosphorylation, suppresses renal cancer cell invasion through AKT-mTOR-STAT3 signaling	Nitta et al. [44]
ELOVL6	Depletion induces a repair-promoting phagocyte phenotype through S1P/PPARγ pathway	Garcia et al. [16]
ELOVL7	Enriched KEGG pathways including PI3K-Akt signaling, lysosome, HIF, Wnt, and AMPK signaling	Risha et al. [49]

PD-1, programmed cell death-1; CTLA-4, cytotoxic T lymphocyte-associated protein-4; HCC, hepatocellular carcinoma; MAPK, mitogen-activated protein kinases; mTOR, mammalian target of rapamycin; Wnt, wint; KEGG, kyoto encyclopedia of genes and genomes; PI3K, phosphatidylinositol 3-kinase; AKT, protein kinase B; STAT3, signal transducer and activator of transcription 3; S1P, sphingosine-1-phosphate; PPARγ, peroxisome proliferator-activated receptor γ

### Specific functions in certain tissues and organs

The superfamily of very-long-chain fatty acid elongases exhibits specific functions in certain tissues and organs, highlighting their tissue-specific roles in physiological processes (Table 4).

**Table 4:** Tissue-specific roles of ELOVL family members in lipid metabolism

Tissue	ELOVL family member	Role and function	References
Brain	ELOVL4	Critical for brain development and function	Ellezam et al. [13]
Skin	ELOVL1	Crucial for skin barrier function, contributes to ceramide synthesis and skin hydration, protection, and barrier integrity	Sassa et al. [53]
Liver	Various ELOVL isoforms	Associated with hepatic lipid metabolism, contribute to lipid droplet formation, triglyceride synthesis, and lipid export	Matsuzaka et al. [34, 35]

#### Brain

Very-long-chain fatty acid elongases, such as ELOVL4, play critical roles in brain development and function. They contribute to the synthesis of very-long-chain polyunsaturated fatty acids, which are essential for neuronal membrane integrity, myelination, and overall brain health [13].

#### Skin

Elongases, such as ELOVL1, are highly expressed in the skin and are crucial for the maintenance of the skin barrier function. They contribute to the synthesis of ceramides and other lipid components, which play a vital role in skin hydration, protection, and barrier integrity [53].

#### Liver

The liver expresses various elongase isoforms, and their activities are closely associated with hepatic lipid metabolism. Elongases contribute to the synthesis and modification of lipids involved in lipid droplet formation, triglyceride synthesis, and lipid export from the liver(Matsuzaka, Kuba, Koyasu, Yamamoto, Motomura, Arulmozhiraja, Ohno, Sharma, Shimura, Okajima, Han, Aita, Mizunoe, Osaki, Iwasaki, Yatoh, Suzuki, & Sone, 2020).

**Fig. 2:**
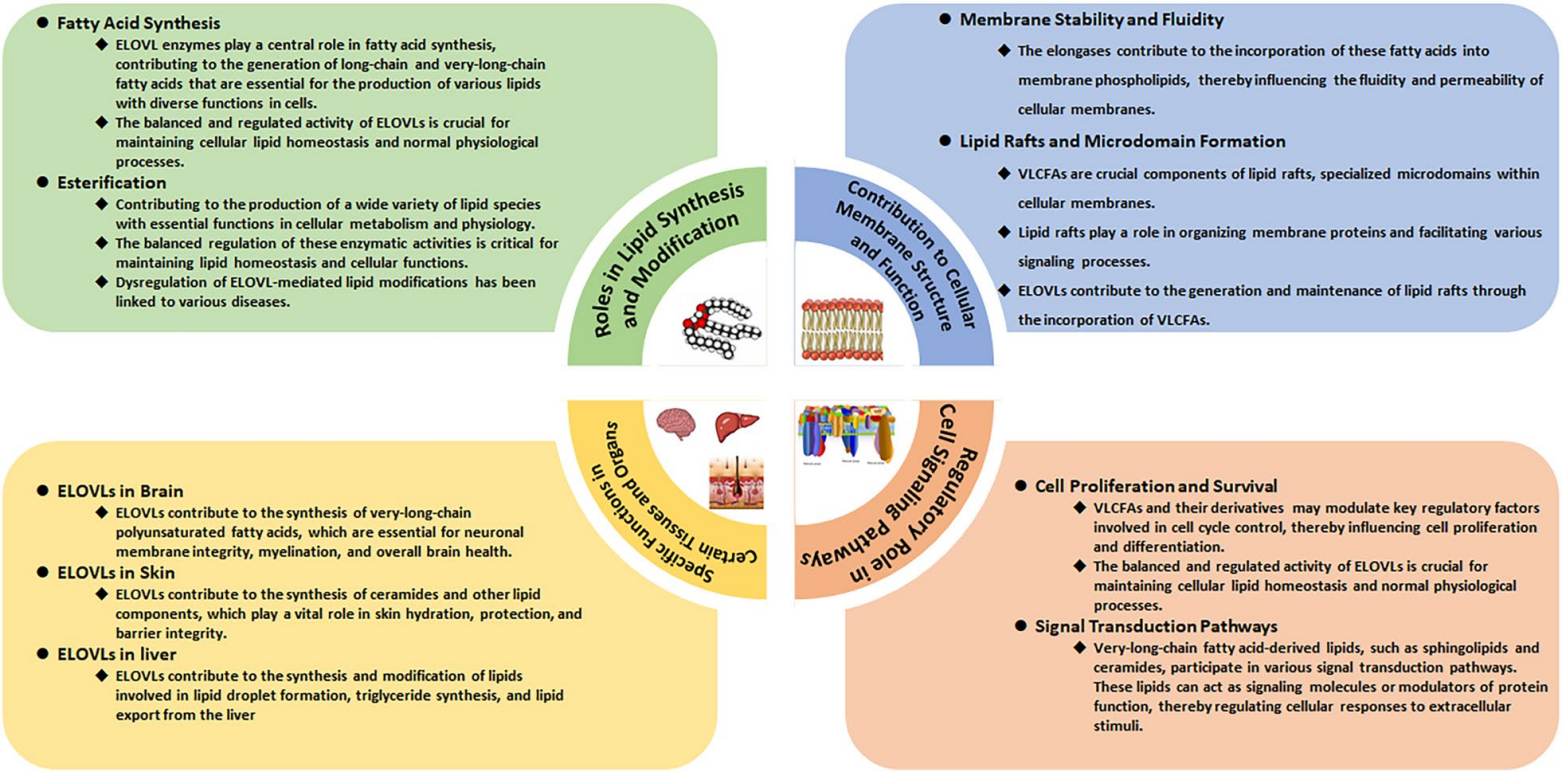
ELOVL family members involve in physiological processes. ELOVLs, very-long-chain fatty acid elongases; VLCFAs, very-long-chain fatty acids

Understanding the role of the ELOVL family in these physiological processes provides valuable insights into their functional significance and their contribution to cellular homeostasis in different tissues and organs (Fig. 2).

## Association of the ELOVL family with diseases

### X-linked adrenoleukodystrophy (X-ALD) and related disorders

The ELOVL1 is closely associated with genetic disorders and metabolic dysregulation. In particular, X-linked adrenoleukodystrophy (X-ALD) and other related diseases have been extensively studied in the context of these enzymes. X-ALD is a genetic disorder characterized by the impaired breakdown and metabolism of VLCFAs. Deficiencies in VLCFA degradation lead to their accumulation in various tissues, including the brain, adrenal glands, and spinal cord. Consequently, individuals with X-ALD may experience neurological symptoms such as cognitive impairment, motor deficits, and behavioral changes. X-ALD can also manifest as adrenal insufficiency, affecting the adrenal glands' ability to produce hormones essential for normal bodily functions [54].

### Role of the ELOVL family in skin disorders, particularly ichthyosis

The ELOVL family plays a significant role in the pathogenesis of various skin disorders, with a particular focus on ichthyosis and related conditions. These disorders are characterized by abnormal skin scaling, dryness, and thickening, resulting from impaired epidermal barrier function.

One prominent example is autosomal recessive congenital ichthyosis (ARCI), a heterogeneous group of disorders caused by mutations in several genes involved in lipid metabolism and epidermal differentiation. Among these genes, certain members of the superfamily of very-long-chain fatty acid elongases, such as ELOVL1 and ELOVL4, have been implicated in ARCI pathogenesis.

The ELOVL1 gene encodes an enzyme responsible for the elongation of fatty acids, including very-long-chain fatty acids, which are crucial for maintaining the epidermal barrier. Mutations in ELOVL1 result in decreased levels of very-long-chain fatty acids and compromised lipid barrier function, contributing to the development of ARCI [41]. ELOVL4, another member of the family, is primarily expressed in the epidermis and plays a vital role in skin homeostasis. Mutations in *ELOVL4* are associated with autosomal dominant ichthyosis and other skin disorders. Loss of ELOVL4 function leads to alterations in epidermal lipid composition, impairing the formation and maintenance of the skin barrier [11].

The disruption of lipid metabolism and epidermal barrier function in ichthyosis highlights the essential role of the superfamily of very-long-chain fatty acid elongases in skin health. These enzymes are involved in the synthesis and elongation of fatty acids necessary for proper barrier formation, hydration, and protection against external factors.

Further investigations into the specific mechanisms by which the superfamily of very-long-chain fatty acid elongases contribute to the pathogenesis of ichthyosis and related skin disorders are warranted. Understanding these mechanisms will facilitate the development of targeted therapeutic approaches aimed at restoring epidermal barrier function and ameliorating the symptoms of these debilitating skin conditions.

### Association of the ELOVL family with metabolic disorders and inflammatory diseases

#### Obesity, type 2 diabetes and cardiovascular diseases

Obesity is characterized by excessive fat accumulation, leading to adverse metabolic effects and increased risk of various diseases. The dysregulation of fatty acid metabolism and lipid synthesis has been implicated in the development of obesity. Studies have shown that altered expression and activity of certain very-long-chain fatty acid elongases, such as ELOVL6, are associated with increased fat storage and obesity in animal models [36, 56].

Type 2 diabetes is characterized by insulin resistance and impaired glucose regulation. Dyslipidemia, particularly elevated levels of VLCFAs, is frequently observed in individuals with type 2 diabetes. Studies have linked alterations in very-long-chain fatty acid elongase activity to insulin resistance and impaired glucose metabolism [29, 46].

Cardiovascular diseases, including atherosclerosis and heart failure, are major causes of morbidity and mortality worldwide. Dysregulated lipid metabolism and inflammation are key contributors to the progression of these diseases. Very-long-chain fatty acid elongases have been implicated in the synthesis of bioactive lipids, such as sphingolipids, which play critical roles in vascular function and inflammation [31].

#### Inflammatory diseases

Inflammatory diseases and immune response regulation are complex processes involving interactions between the immune system and various cells and tissues. The ELOVL family has emerged as an important player in these pathways, influencing inflammatory responses and immune cell function.

Inflammatory bowel disease (IBD), including Crohn's disease and ulcerative colitis, is characterized by chronic inflammation of the gastrointestinal tract. Dysregulated lipid metabolism and altered production of pro-inflammatory lipid mediators have been implicated in IBD pathogenesis. ELOVLs are involved in the synthesis of inflammatory lipid mediators, such as leukotrienes and prostaglandins, which contribute to the inflammatory process in IBD [40].

Autoimmune diseases, including rheumatoid arthritis and multiple sclerosis, result from dysregulated immune responses against self-antigens. ELOVLs are involved in immune cell activation and regulation. Dysfunctional elongase activity may contribute to aberrant immune cell function and promote the development of autoimmune responses [3].

**Fig. 3:**
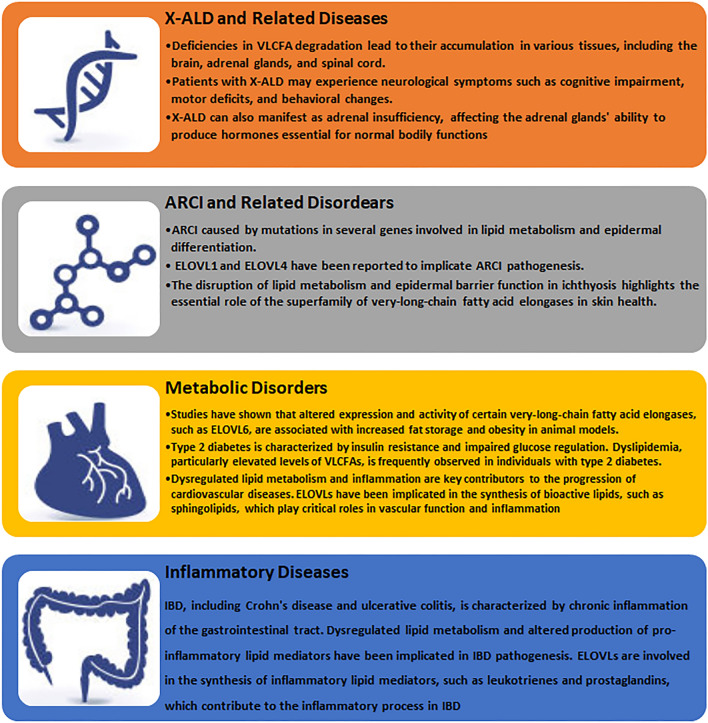
Role of ELOVL Family Members in Non-neoplastic Diseases. X-LAD, X-linked adrenoleukodystrophy; ARCI, autosomal recessive congenital ichthyosis; ELOVLs, very-long-chain fatty acid elongases; VLCFAs, very-long-chain fatty acids

Understanding the role of the superfamily of very-long-chain fatty acid elongases in metabolic disorders and inflammatory diseases has significant implications for developing novel therapeutic strategies. Targeting these enzymes and their associated lipid signaling pathways may provide potential avenues for treating these complex and prevalent diseases. However, further research is needed to unravel the exact mechanisms and regulatory pathways involved, paving the way for more effective and tailored therapeutic interventions (Fig. 3).

### ELOVLs and cancer

Cancer cells undergo metabolic changes to facilitate their survival, growth, and ability to spread. Changes in fatty acid metabolism within cancer cells have garnered growing interest [7]. ELOVLs, by influencing fatty acid metabolism, have emerged as independent prognostic indicators for cancer prognosis. They have also become valuable biomarkers and potential targets for clinical diagnosis and the treatment of diverse cancer types.

ELOVL1, the first member of the ELOVL family, has been implicated in several cancer types, including breast cancer. It promotes cancer cell proliferation and migration through alterations in lipid metabolism, affecting membrane lipid composition and pro-tumorigenic lipid mediator production [69]. ELOVL2 has been linked to cancer progression in colorectal cancer and prostate cancer [17, 38]. It influences cancer cell proliferation and migration by modulating membrane lipid composition and regulating signaling pathways involved in cancer progression. In colorectal cancer, ELOVL2 expression is associated with tumor growth and metastasis [38]. ELOVL3 exhibited upregulation following the overexpression of BRG1, while it was downregulated after BRG1 knockdown in prostate cancer cells. Subsequent analysis unveiled that BRG1 plays a role in promoting the migration and invasion of prostate cancer cells. This is achieved by the interaction of BRG1 with p300, which leads to an epigenetic modulation of RORγ-dependent ELOVL3 transcription [67]. ELOVL4 has been implicated in cancer angiogenesis, particularly in gastric and breast cancer [1, 66]. It promotes the synthesis of pro-angiogenic lipids, supporting tumor vascularization and growth. ELOVL4 expression is repressed by MYCN Proto-Oncogene (MYCN) associated with good prognosis in glioblastoma patients [51]. ELOVL5 is involved in various cancer types, including renal cancer and breast cancer [43, 44]. It contributes to cancer cell proliferation and migration by regulating the synthesis of VLCFAs involved in cancer cell membrane dynamics. In breast cancer, ELOVL5 down-regulation is associated with increased tumor growth and metastasis through a lipid-droplet accumulation-mediated induction of TGF-β receptors [23]. ELOVL6 is implicated in metabolic reprogramming in several cancer types, such as colorectal cancer and hepatocellular carcinoma [58, 61]. It promotes lipogenesis and facilitates cancer cell adaptation to nutrient availability. In hepatocellular carcinoma, knockdown of Elovl6 in HCC cells reduced cell proliferation and Akt activation, as well as sensitivity to fatty acids. Inhibition of Elovl6 reduced tumor growth and prolonged survival in mice bearing tumors [58]. ELOVL7 plays a crucial role in cancer cell migration and metastasis in prostate cancer, which involves in prostate cancer growth and survival through the metabolism of SVLFAs saturated very-long-chain fatty acids (SVLFA, C20:0 approximately) and their derivatives [59] (Table 5).

**Table 5:** Involvement of ELOVL family members in cancer progression and tumor biology

ELOVL family member	Implication in cancer	Role in cancer	References
ELOVL1	Implicated in breast cancer	Promotes cancer cell proliferation and migration through alterations in lipid metabolism, affecting membrane lipid composition and pro-tumorigenic lipid mediator production	Zhang et al. [69]
ELOVL2	Linked to colorectal and prostate cancer	Influences cancer cell proliferation and migration by modulating membrane lipid composition and regulating signaling pathways involved in cancer progression	Hu et al. [17], Monirujjaman et al. [38]
ELOVL3	Implicated in prostate cancer	Promotes cancer cell migration and invasion by epigenetically modulating ELOVL3 transcription	Yang et al. [67]
ELOVL4	Implicated in gastric and breast cancer	Promotes the synthesis of pro-angiogenic lipids, supporting tumor vascularization and growth; repression by MYCN associated with good prognosis in glioblastoma patients	Agostini & Melino [1], Yang et al. [66] and Rugolo et al. [51]
ELOVL5	Involved in renal and breast cancer	Contributes to cancer cell proliferation and migration by regulating the synthesis of VLCFAs involved in cancer cell membrane dynamics; down-regulation associated with increased tumor growth and metastasis	Kieu et al. [23]
ELOVL6	Implicated in colorectal cancer and hepatocellular carcinoma	Promotes lipogenesis and facilitates cancer cell adaptation to nutrient availability; knockdown reduces cell proliferation and AKT activation, as well as sensitivity to fatty acids	Su et al. [58], Tian, Li, & Ge [61]
ELOVL7	Crucial in prostate cancer	Involved in prostate cancer growth and survival through the metabolism of SVLFAs and their derivatives	Tamura et al. [59]

MYCN, N-myc proto-oncogene protein; VLCFA, very long chain fatty acid; AKT, protein kinase B; SVLFA, saturated very-long-chain fatty acid

The ELOVL family of enzymes exerts diverse effects on cancer biology through distinct mechanisms, making them potential targets for personalized cancer therapies. Elucidating the roles of individual ELOVL isoforms in different cancer types provides valuable insights for developing targeted therapies and advancing precision medicine strategies for cancer patients.

## Conclusion

Throughout this review, we have provided a comprehensive overview of the various members of the ELOVL family. Each isoform has distinct structural and functional characteristics, contributing to lipid metabolism and cellular processes in unique ways. From ELOVL1 to ELOVL7, these enzymes have emerged as critical players in lipid biosynthesis and signaling, influencing cell membrane dynamics, cellular responses to stress, and various physiological functions.

The significance of the ELOVL family in both normal physiology and disease development cannot be understated. These enzymes play vital roles in lipid homeostasis, membrane composition, and cellular functions across multiple tissues and organs. Dysregulation of ELOVLs has been implicated in various diseases, including metabolic disorders, skin diseases, neurodegenerative conditions, and cancer. Understanding their precise roles in these processes can shed light on potential therapeutic targets and diagnostic biomarkers for diverse pathologies.

The study of the ELOVL family is a rapidly evolving field with promising future prospects. Further investigation into the molecular mechanisms and regulatory networks governing ELOVL function will deepen our understanding of lipid biology and its impact on human health. The identification of isoform-specific inhibitors and activators may lead to novel therapeutic strategies in managing metabolic diseases and cancer. Additionally, the elucidation of ELOVL-based biomarkers may facilitate early disease detection and patient stratification for personalized treatment approaches.

In conclusion, the ELOVL family of enzymes represents a crucial nexus between lipid metabolism and cellular physiology, impacting various biological processes with implications in health and disease. From their role in lipid elongation to their contributions in cancer biology and metabolic regulation, ELOVLs have emerged as fascinating molecular targets with significant therapeutic potential. As research in this field progresses, the insights gained from the study of the ELOVL family will undoubtedly pave the way for innovative strategies in disease management and personalized medicine.

## Data Availability

Not applicable.
